# Isolation by distance versus landscape resistance: Understanding dominant patterns of genetic structure in Northern Spotted Owls (*Strix occidentalis caurina*)

**DOI:** 10.1371/journal.pone.0201720

**Published:** 2018-08-02

**Authors:** Mark P. Miller, Raymond J. Davis, Eric D. Forsman, Thomas D. Mullins, Susan M. Haig

**Affiliations:** 1 U.S. Geological Survey, Forest and Rangeland Ecosystem Science Center, Corvallis, Oregon, United States of America; 2 U.S. Department of Agriculture Forest Service, Pacific Northwest Region, Corvallis, Oregon, United States of America; University of South Carolina, UNITED STATES

## Abstract

Landscape genetics investigations examine how the availability and configuration of habitat influence genetic structure of plants and animals. We used landscape genetics to evaluate the role that forest connectivity plays in determining genetic structure of the federally-threatened Northern Spotted Owl (*Strix occidentalis caurina*) using genotypes of 339 Northern Spotted Owls obtained for 10 microsatellite loci. Spatial clustering analyses identified a distinct genetic cluster at the southern extent of the region examined. This cluster could not be linked to landscape connectivity patterns and suggested that post-Pleistocene processes were involved with its development rather than contemporary landscape configuration. We also compared matrices of pairwise inter-individual genetic distances with resistance distances derived from a circuit-theory based framework. Resistance distances were obtained for an idealized raster map that reflected continuous unimpeded dispersal habitat across the landscape along with five empirically-derived raster maps reflecting the 1870’s, 1940’s, 1986, 1994, and 2012. Resistance distances from the idealized map served as surrogates for linear geographic distances. Relative to idealized conditions, resistance distances were ~250% higher in the 1940’s and ~200% higher from 1986 onward. Resistance distances from the 1870’s were ~40% higher than idealized conditions. Inter-individual genetic distances were most highly correlated with resistance distances from the idealized map rather than any of the empirical maps. Two hypotheses explain our results. First, our results may reflect temporal lags between the onset of large-scale habitat alterations and their novel effects on genetic structure in long-lived species such as Northern Spotted Owls. Second, because Northern Spotted Owls disperse over long distances, our results may indicate that forest habitat has never been sufficiently fragmented to the point where connectivity was disrupted. The second hypothesis could indicate that forest management practices mandated by the Northwest Forest Plan succeeded with one of its primary goals. However, our results do not represent a complete portrayal of the status of Northern Spotted Owls given detection of significant population declines and bottlenecks in other studies. Future investigations based on computer simulations may help distinguish between hypotheses.

## Introduction

Landscape genetics, a fusion of population genetics and landscape ecology [1], aims to identify specific landscape attributes that influence patterns of gene flow, genetic drift, or selection in a given taxon. Landscape-scale patterns of habitat quality and its configuration is emphasized [2–3], in particular due to its influence on organismal movement and gene flow. Gene flow is an especially important process, as it reflects patterns of connectivity and movement of individuals among populations and can help determine if specific landscape regions are more isolated than others [4]. A deeper understanding of landscape-scale population connectivity patterns may provide resource managers with better information for designing habitat reserve networks and prioritizing habitat restoration programs that facilitate connectivity or for implementing more focused strategies to help protect and manage isolated populations.

**Fig 1:**
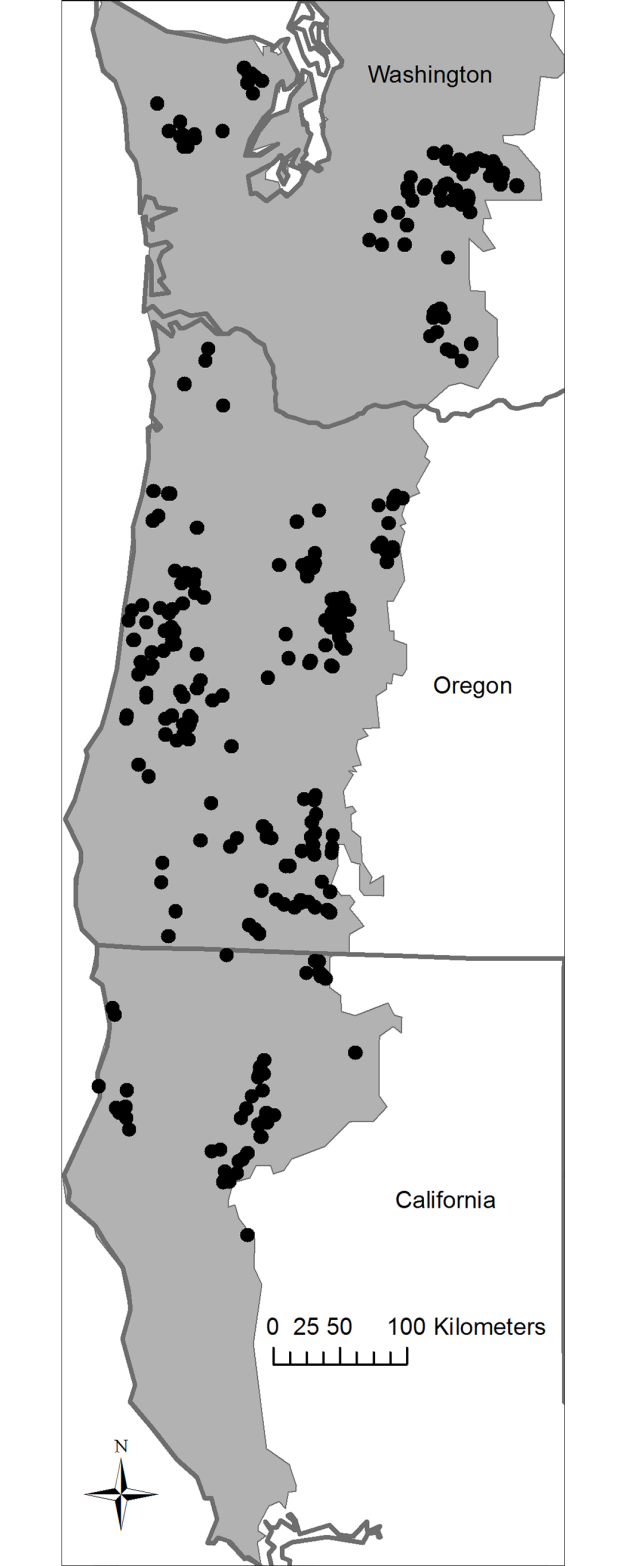
Map highlighting sampling locations of 339 Northern Spotted Owls used in this investigation. Shaded areas indicate the Northern Spotted Owl’s range.

In this study, we applied landscape genetic techniques to investigate how forest habitat and configuration are associated with genetic differentiation patterns across the range of Northern Spotted Owls (*Strix occidentalis caurina*). Northern Spotted Owls occur along the northwest coast of North America from southwestern British Columbia to central California [5] (Fig 1). One of three spotted owl subspecies, the Northern Spotted Owl was listed as threatened under the U.S. Endangered Species Act in 1990 as a consequence of population declines. The primary factor influencing the northern spotted owl population at the time of listing was habitat loss [6], but competition with non-native, invading Barred Owls (*S*. *varia*) has more recently become a major contributing factor [7–8]. Since its listing, the taxon has been the subject of extensive research and monitoring to identify habitat requirements and demographic trends. Despite active management under the Northwest Forest Plan [9], Northern Spotted Owl populations have declined by an average of 3.8% per year between 1985 and 2013 across the federal, state, and privately-owned lands that they inhabit [7]. Likewise, nesting and roosting forest cover decreased 3.4% between 1993 and 2012, whereas forest cover types conducive to Northern Spotted Owl dispersal decreased by 2.3% during that time [10].

Previous assessments of landscape genetics patterns in Northern Spotted Owls [11] primarily relied on the “Genetic Landscape Shape” procedure [12] in the program Alleles in Space [13], which provided a qualitative graphical depiction of landscape-scale spatial genetic structure patterns. Analyses using GENELAND [14] suggested that there were no spatial genetic clusters across the landscape [11], although Miller et al. [15] later identified a small population in the southern part of the owl’s range that forms a distinct genetic cluster, possesses a unique mtDNA lineage, and has a high proportion of hybrids between Northern Spotted Owls and California Spotted Owls. In this study, we applied more refined and explicit statistical tests that evaluated associations between genetic structure patterns and landscape-level connectivity as quantified by patterns of isolation by distance and isolation by resistance. Under isolation by distance, genetic differences of individuals on a landscape are expected to increase with increasing straight-line, geographic distances between landscape locations. By contrast, under an isolation by resistance process, all possible pathways between geographic locations can be taken into consideration along with quantitative information on habitat quality and its suitability for promoting organismal movement [16]. Simulation studies and empirical investigations show that resistance distances generally outperform simple geographic distances in explaining variation in genetic differentiation patterns across a landscape [16, 17]. Overall, we calculate and compare measures of resistance distances based on idealized, historical, and contemporary forest composition to provide a more rigorous assessment of the effects of landscape structure on genetic differentiation patterns in Northern Spotted Owls. Results of our analyses provide insights towards one facet of federal land management practices used to preserve forest habitat structure in the Pacific Northwest.

## Methods

### Genetic data

We analyzed a data set comprised of 10 nuclear microsatellite loci [11, 15] that were genotyped in 339 Northern Spotted Owls sampled from throughout the majority of the subspecies’ range since the mid-1990’s (Fig 1). Of the 339 individuals examined, 304 were based on data from non-hybrid individuals produced by Funk et al. [11],. Comparable data from an additional 35 non-hybrid Northern Spotted Owl individuals identified by Miller et al. [15] in northern California between 2012 and 2015 were also included. When new samples were genotyped for the more recent study [15], samples from earlier studies [11] were run concurrently to ensure that no allelic shifts were observed that could be attributed to laboratory variation and analyses at different points in time.

### Identifying spatial genetic clusters

Previous analyses [11] based on GENELAND [14] suggested that there was no overt spatial genetic structure across the range of Northern Spotted Owls. However, a more recent study that included additional samples from more southern parts of the taxon’s range identified a separate genetic cluster that possessed a large proportion of individuals identified as hybrids between Northern Spotted Owls and California Spotted Owls (*S*. *o*. *occidentalis*) [15]. Given our focus specifically on Northern Spotted Owls in this investigation, we repeated the previous GENELAND analyses and included the more recent non-hybrid samples described above to determine if the southern genetic cluster can be discerned in the absence of hybrid individuals. Analyses were performed using GENELAND version 4.0.8 with the uncorrelated allele frequency model. The potential number of genetic clusters was allowed to vary between one and 10, and analyses were based on 5 independent runs, each based on 5*10^5^ Markov-Chain Monte-Carlo replicates and a thinning interval of 500 (1000 saved replicates). The first 200 saved replicates were discarded as burnin in post-processing steps. Geneland was also used to quantify the extent of differentiation of any detected clusters using the standard measure F_ST_ to reflect the magnitude of differentiation.

### Quantifying genetic distances, geographic distances and landscape resistance

**Fig 2:**
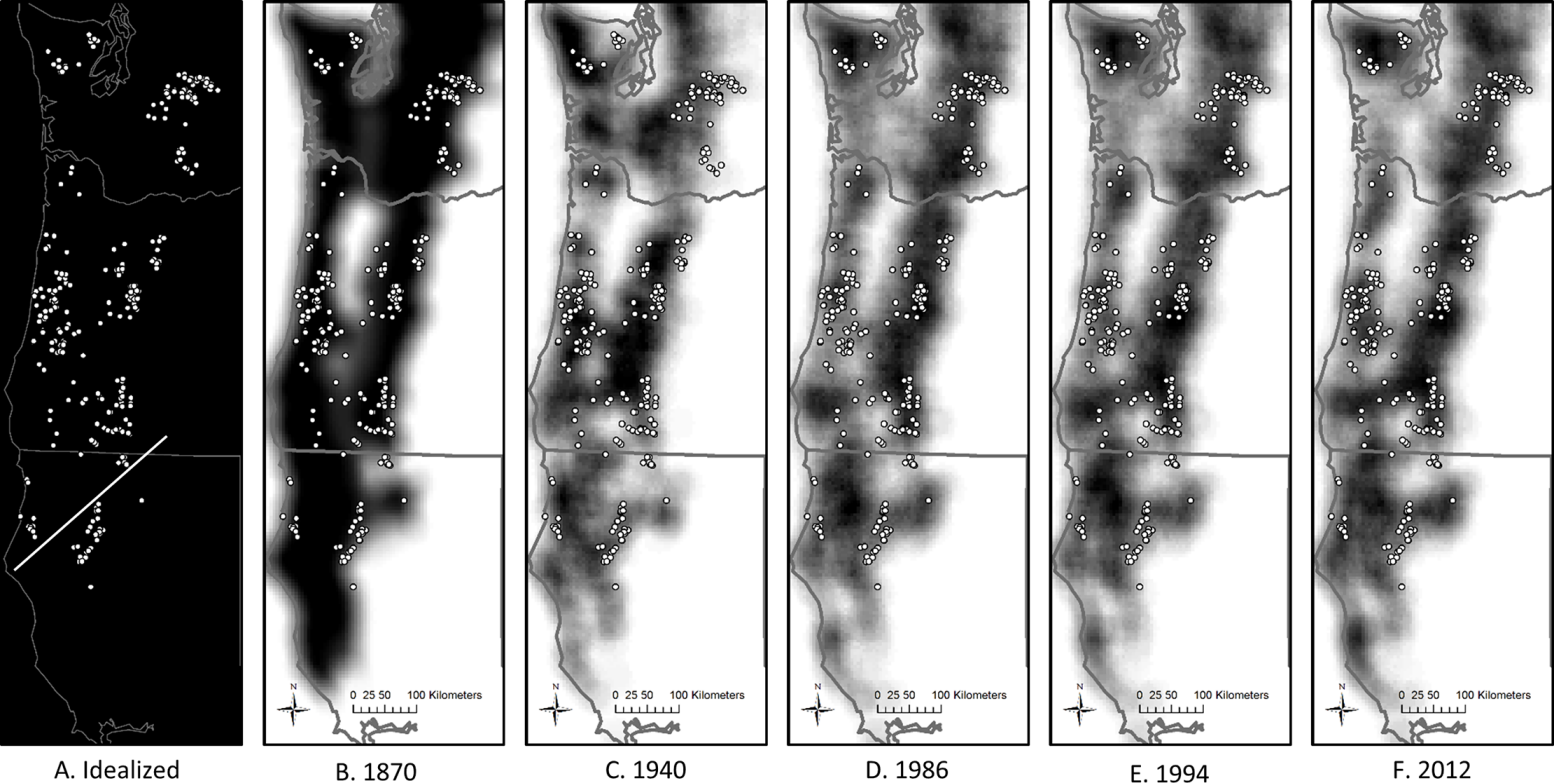
Map representations of six conductance surfaces overlaid with genetic samples collected that were used to examine genetic structure patterns in Northern Spotted Owls. Panel A represents an idealized conductance surface where all regions of the landscape were equally conducive to Spotted Owl dispersal. Panels B through F represent habitat quality determined for points in time spanning 1870 to 2012, with darker pixels representing areas with greater landscape conductance. The straight line drawn on panel A indicates a spatial divide separating sample sets that was resolved using GENELAND [14].

Matrices of inter-individual genetic and geographic distances were calculated for all pairwise combinations of individuals with the program Alleles in Space [13]. Genetic distances were quantified based on the method of Nei et al. [18]. We used Circuitscape version 4.0 [19] to quantify landscape resistance distances between all pairs of sampled individuals. Resistance distances make use of GIS-based raster maps and circuit theory-based calculations to quantify the degree of connectivity among different landscape locations [16, 17]. Raster maps used for analyses were based on six models representing landscape conductance (the reciprocal of resistance) for Northern Spotted Owls under a simple conceptual framework (Fig 2A) or over different time frames encompassing the past ~150 years (Fig 2B–2F). Northern Spotted Owls primarily use a matrix of forested habitat for dispersal [20], therefore, our conductance surfaces incorporated information that reflected forest condition. Under the first model (Fig 2A), we considered a hypothetical conductance surface where all locations on the landscape were equally (and maximally) conducive to owl dispersal and movement. The surface was represented as a raster layer where all raster cells were set to 100, reflecting the greatest conductance values used in this study. Use of this approach allows for derivation of a resistance distance matrix that is highly correlated with geographic distances, but is more easily compared to resistance distances calculated from other raster maps that more realistically portray actual habitat conditions [21]. The second conductance surface was based on maps of North American forest density in 1870 by William H. Brewer [22]. The Brewer maps present information that describe woodland density (acres per square mile) and are available in digital form [23–24]. We assumed that raster cells with higher woodland density values were more conducive to individual movement in our analyses. Third, we used a conductance surface representing dispersal habitat of Northern Spotted Owls in the 1930’s-1940’s as described by Davis and Lint [25], which was based on aerial photography and habitat surveys from the Pacific Northwest [26–28]. Finally, we used three additional conductance surfaces to reflect dispersal habitat for 1986, 1994, and 2012 using criteria from Thomas et al. [29]. These maps were generated as described in Davis et al. [10,30], and incorporated information on forest cover types derived from Landsat imagery associated with each year of interest. Genetic data, spatial information, and raster maps used in all analyses are available online (https://doi.org/10.5066/F7J67FVW).

All conductance surfaces were scaled to range between 0 (poor dispersal habitat) and 100 (excellent dispersal habitat) and were produced at a 5-km spatial resolution (5 km raster cells). This distance is well below the median juvenile dispersal distances described in Forsman et al. [20] (Median natal dispersal distances were 14.6 km for banded males, 13.5 km for radio-marked males, 24.5 km for banded females, and 22.9 km for radio-marked females). Analyses that quantified landscape resistance distances between sampling locations of owls from genetic analyses were performed with Circuitscape 4.0 [19] in pairwise mode using all eight connections among neighboring raster cells.

We identified the landscape resistance or spatial-based distance matrix that was most highly correlated with pairwise inter-individual genetic distances. Mantel tests [31] with 5000 permutations were performed using program *zt* [32] to determine the correlation coefficient (*r*) and associated *P*-value for each analysis. To help clarify our results, we also used *zt* to evaluate correlations between each of the 6 resistance distance matrices derived for our dispersal habitat maps (Fig 2) and the matrix of pairwise geographic distances.

We compared the matrix of resistance distances from each time point to the congruent matrix of resistance distances produced from analyses of the idealized habitat map portrayed in Fig 2A. Comparisons were designed to identify time points that deviated the most and least from idealized conditions and determine if any temporal trends in resistance distance patterns existed. Differences between resistance distance matrices were calculated as Δ*r*, where

$$\Delta r=100\times \frac{\left[\sum_{i=2}^{n}\sum_{j=1}^{i-1}(r_{i,j}^{temporal}-r_{i,j}^{ideal})/r_{i,j}^{ideal}\right]}{n\times (n-1)/2}$$

and *r*^*ideal*^ is the matrix of pairwise resistance distances derived from the idealized habitat map, *r*^*temporal*^ is a resistance distance matrix derived from one of the specific time points investigated in this study, *n* is the number of individuals examined, and the quantity *n*×(*n*−1)/2 is the number of pairwise resistance distances. Δ*r* essentially reflects the average percent difference of elementwise resistance distances between the two matrices being compared.

## Results

Spatial genetic clustering analyses consistently identified 2 distinct genetic clusters among each of the five analysis replicates performed. Similar to that observed in prior studies [14], the clusters distinguish between owls from the majority of the Northern Spotted Owl’s range and those from the extreme southern part of the taxon’s range (Fig 2A; F_ST_ = 0.036 between clusters).

**Fig 3:**
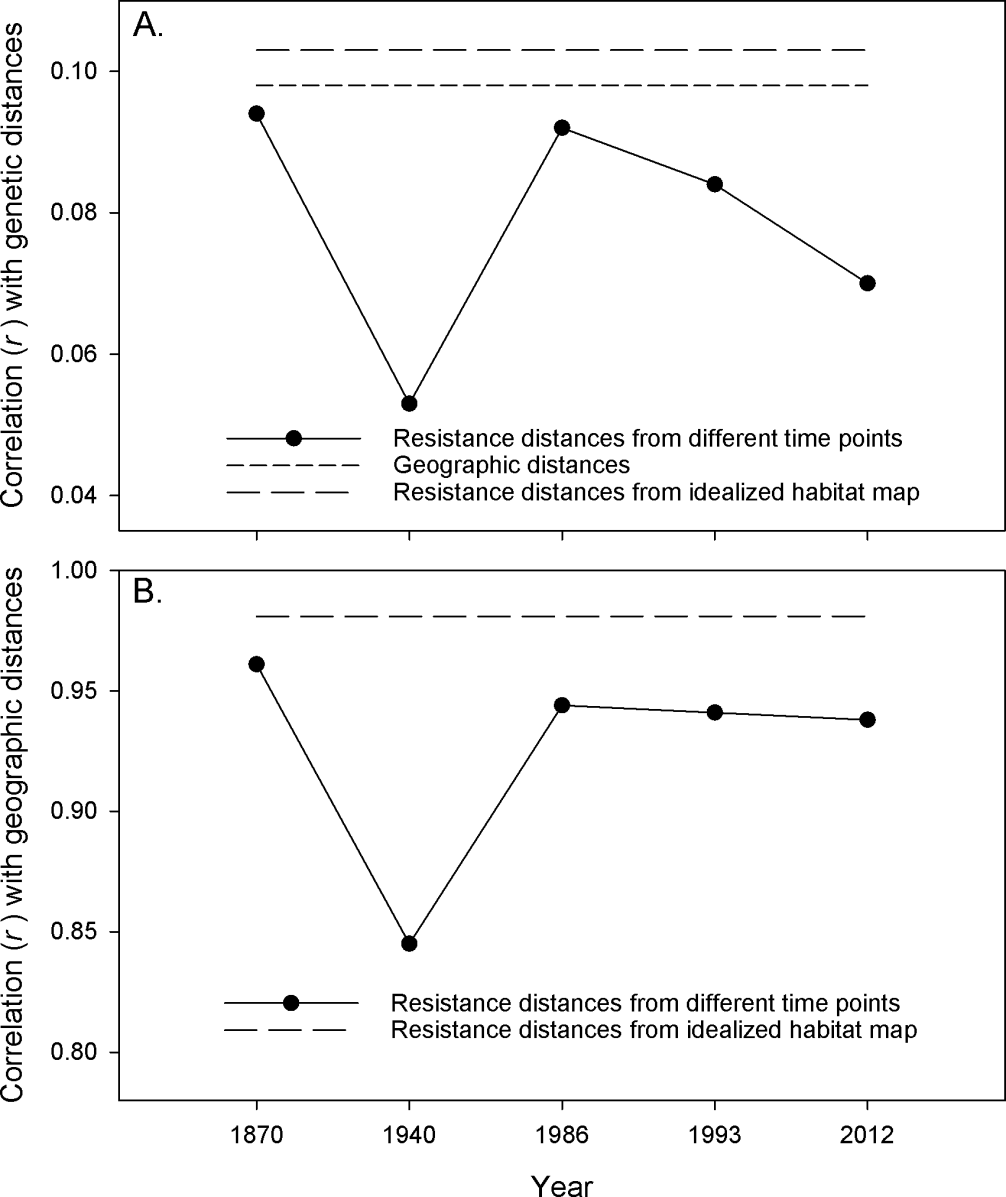
Correlations between different genetic, geographic, and landscape resistance distance matrices. A. Correlations between pairwise genetic differentiation measures derived from contemporary Northern Spotted Owl samples and congruent resistance distance or geographic distance matrices. Resistance distances were derived from analyses of raster maps that represented Northern Spotted Owl dispersal habitat at different time points (Fig 2B–2F) or for an idealized hypothetical habitat map where all landscape locations were equally (and maximally) suitable for owl dispersal (Fig 2A). Closed circles represent correlations from analyses involving resistance distances associated with forest habitat from five time points over the past ~150 years. Resistance distances from the idealized habitat map (Fig 2A) and geographic distances between points are independent of time; therefore correlation coefficients based on analyses that included these matrices are represented by horizontal dashed lines. All correlations are highly significant (P < 0.005). B. Correlations between pairwise geographic distances and different resistance distance matrices obtained from analyses of maps of Northern Spotted Owl dispersal habitat for different time points (Fig 2B–2F) or an idealized Northern Spotted Owl habitat map (Fig 2A). Closed circles represent correlations from analyses based on data from different time points. The idealized habitat map is independent of time and is therefore represented by a horizontal line. All correlations are highly significant (P < 0.005).

Our analyses revealed low, but highly significant (P < 0.005), correlations between pairwise inter-individual genetic distances and all measures of landscape connectivity (geographic distances and resistance distances; Fig 3A). Resistance distances associated with the idealized habitat map (Fig 2A) yielded the highest correlation (*r* = 0.103), whereas distances associated with forest condition from the 1940’s produced the lowest (*r* = 0.053). The correlation between genetic distances and simple geographic distances was on par with that observed for the idealized habitat (*r* = 0.098). Each resistance distance matrix was also highly correlated with geographic distances (Fig 3B). The idealized resistance distances demonstrated the strongest correlation (*r* = 0.981) and the 1940’s resistance distances producing the lowest correlation (*r* = 0.845; Fig 3B).

**Fig 4:**
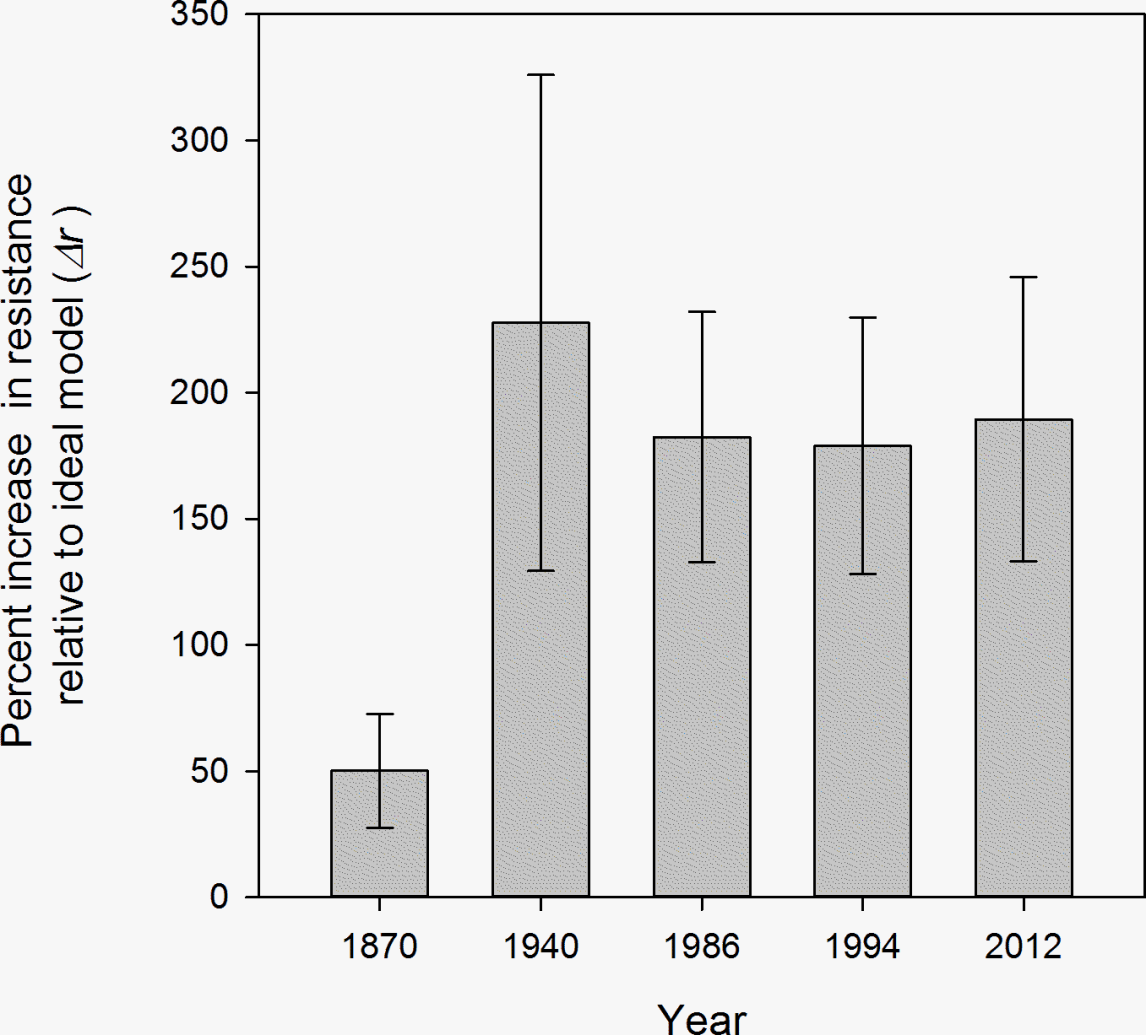
Percent differences in pairwise resistance distances for comparisons of five time points (Fig 2B–2F) relative to resistance distances derived from an idealized dispersal habitat map for Northern Spotted Owls (Fig 2A). Means and standard deviations are presented.

Resistance distances from the 1940’s landscape were 228% greater than those from the idealized conductance surface, whereas forest condition in the 1870’s was most similar (Fig 4). Resistance distances from 1986, 1994, and 2012 were ~180% greater than idealized resistance distances and showed minimal indication of change over that time span.

## Discussion

The Northwest Forest Plan [9] was developed in response to conflicts among forest management practices, timber harvest, and legal requirements to provide habitat for native species of plants and animals, including threatened and endangered species. Habitat for Northern Spotted Owls was a primary consideration when the plan was initiated, which placed approximately 78% of the 10 million hectares of federal lands into reserves or administrative units that precluded or restricted logging [33–35]. Although forest succession has improved habitat in many areas since the implementation of the Northwest Forest Plan, there has been a 1.5% net decrease in spotted owl nesting and roosting forest cover types on federal lands within the area of the Northwest Forest Plan since 1993, with large forest wildfires accounting for most of the loss [10]. However, this rate of change is far less than that estimated for the period preceding plan implementation, when rates of loss were on the order of 5% per decade in the 1940’s [10].

In this study, we used landscape genetic analyses to better understand the role that forest connectivity plays in determining genetic connectivity for Northern Spotted Owls across their range. We illustrated that the separate genetic cluster identified in a previous study [15] was not due to the presence of hybrids, since only non-hybrid individuals were included in our current analyses. Miller et al. [15] hypothesized that landscape genetic studies could be used to better understand the basis for this differentiation pattern, however, our analyses from this study suggested that owl dispersal habitat configuration could not be conclusively linked to genetic differentiation. Genetic discontinuities can in some instances be formed in the absence of clear barriers to dispersal [36], and multiple empirical studies from the Pacific Northwestern United States have revealed genetic discontinuities that may reflect post-Pleistocene processes rather than contemporary landscape effects [37–39]. Miller et al. [15] also showed that many owls from this southern cluster possess haplotypes from a distinct, previously undocumented mitochondrial DNA clade. This finding is consistent with the primary influence of historical rather than contemporary processes in producing the two separate genetic clusters.

Resistance distances, as derived in this study, are theoretically expected to more accurately reflect genetic distance patterns across a landscape than simple geographic distances [16, 40]. Multiple empirical studies have demonstrated the utility of the circuit-theory based approach that is used to derive resistance distances [17, 41–42]. However, in this study, none of the empirically-derived conductance surfaces produced resistance distances that were most highly correlated with genetic distances (Fig 3A). Instead, resistance distances derived for an idealized conductance surface (Fig 2A) produced the strongest association with genetic differentiation patterns. We note that resistance distances derived from this idealized conductance surface are essentially a surrogate for geographic distances [21], as evidenced by their strong overall correlation with simple geographic distances in our Mantel tests (Fig 3B).

Data used to derive the conductance surfaces from this study varied in the original methods used to develop them (ground surveys, aerial photography, and satellite imagery). Of the time points examined, data from 1986 onward should be considered most accurate and robust due to its reliance on contemporary, high-resolution approaches [10, 30]. Data from 1870 were based on generalized ground-based surveys conducted not long after the United States acquired the Oregon Territory from Britain (1846) and California from Mexico (1848). Of the empirically-derived conductance surfaces, the 1870’s resistance distances were most closely correlated with simple geographic distances (Fig 3) and had the lowest percent change relative to resistance distances calculated from the idealized conductance surface (Fig 4). We suggest that these quantitative measures are likely robust given that relatively little large-scale modifications to the landscape had occurred in the western U.S. during that time and indicate that the conductance surface provides a reasonable representation of forest condition for our analyses. Data from the 1940’s showed the lowest association with contemporary genetic differentiation patterns. Resistance distances calculated from the 1940’s data were the least correlated with genetic differentiation patterns (Fig 3) and were substantially greater than resistance distances derived from the idealized conductance surfaces (Fig 4). At this time, we are unable to rule out the possibility that these historical data are of lower quality and that the smaller association with genetic structure patterns is an artefact of data quality issues. However, the 1940’s data were based on a combination of aerial photography and intensive ground-based surveys that were initiated in response to the 1928 McSweeney-McNary Forestry Research Act, which directed the U.S. Agriculture Secretary to make thorough inventories of forest resources throughout the country [27]. Based on quantitative comparisons of these maps with more modern maps derived for 1994 and 2002, forest habitat across the Northern Spotted Owl’s range has changed in varying ways (increasing, decreasing, or unchanged) in different regions since the 1940’s [25]. Given that these surveys were part of early systematic efforts to obtain detailed forest inventory information in the U.S., they likely provide a realistic portrayal of forest condition from that time period and indicate that connectivity for Northern Spotted Owls was reduced in the 1940’s relative to current conditions.

We suggest that there are two possible non-exclusive hypotheses that explain our results. First, our findings may indicate the presence of a temporal lag between landscape condition, as reflected by the empirical raster maps, and genetic structure patterns in Northern Spotted Owls. Temporal lags in genetic structure patterns are expected following changes in landscape connectivity, as many generations may be necessary for genetic drift to alter allele frequencies in populations [43]. Thus, *a priori*, we would not necessarily expect to identify the strongest correlation between genetic distances and resistance distances from the 2012 conductance surface, or possibly even the 1986 or 1994 conductance surface, given that samples were collected in the mid 1990’s or later and that Northern Spotted Owls have a generation time of ~7–12 years [44, 45]. Under this hypothesis our data suggest that Northern Spotted Owl genetic structure is currently reflecting habitat conditions from the 1870’s (Fig 2B) or earlier, prior to the widespread landscape modifications and logging practices that have subsequently occurred throughout the Pacific Northwest. A similar finding came from a landscape genetic study of Pfrimer’s Parakeet (*Pyrrhura pfrimeri*) from the Paranã River Basin in Brazil, where genetic structure patterns were most highly correlated with landscape connectivity patterns from the 1970’s (the earliest time points examined)[41] prior to the large-scale deforestation and agricultural development that has since occurred in the region [46]. In contrast to patterns of genetic connectivity, demographic impacts of habitat fragmentation may be detected quickly, as evidenced by detection of recent population bottlenecks [47] and continued population declines in Northern Spotted Owls across most of their range that are associated with habitat availability [7]. Thus, if this hypothesis is correct, parameters more closely associated with demographic processes would provide a better reflection of the effects of habitat condition on Northern Spotted Owls.

Second, our analyses may indicate that forest habitat suitable for dispersal in the Pacific Northwest has not yet been sufficiently fragmented to the point where genetic structure patterns of Northern Spotted Owls have been influenced. This hypothesis is particularly tenable for long-lived species with high dispersal abilities such as Northern Spotted Owls. Juvenile Northern Spotted Owls can disperse in excess of 100 km [20, 48], with over 8% of dispersal events exceeding 50 km [49]. These long distance dispersal events could allow Northern Spotted Owls to traverse or fly around potential barriers to dispersal. For example, the Willamette Valley, Umpqua Valley, and Rogue River Valley possess limited spotted owl habitat and are generally avoided by dispersing spotted owls [20] (S1 Fig). However, instances of dispersal across these regions have been documented [20, 49], suggesting that sufficient gene flow occurs around these areas such that genetic connectivity is maintained. Thus, despite the fact that dispersal habitat is currently more fragmented and resistant to movement than it was in the 1800’s (Fig 3), our results may indicate some degree of resilience of Northern Spotted Owls to habitat fragmentation and its effects on population genetic processes. If this hypothesis is correct, then our results may resemble those from Harrison et al. [50], who identified no detectable genetic effects of habitat fragmentation in four different woodland bird species. From a conservation perspective, Harrison et al. [50] postulated that it may be more important to emphasize an increase in landscape extent over increasing intervening habitat given the apparent high degree of connectivity identified in their study system. We note, however, that despite the appearance of connectivity reflected by our analyses, Northern Spotted Owls continue to demonstrate population declines across their range [7]. These patterns may therefore indicate in part that dispersal habitat can be of lower quality than the habitat required for nesting and roosting in Northern Spotted Owls.

If this second hypothesis is correct, one additional interpretation of our findings is that the federal forest management practices mandated by the Northwest Forest Plan have, to date, been successful in fulfilling one of its many goals: maintaining genetic connectivity for Northern Spotted Owl throughout their range. Despite the taxon’s potential for long distance dispersal, there is likely to be a lower limit threshold for habitat connectivity that, if exceeded, would have negative consequences for population connectivity. Under the second hypothesis, our results may indicate that this threshold has not been exceeded, although we do not know the upper limit of habitat fragmentation that can be tolerated in this system before connectivity is affected or if that threshold would have been approached if the Northwest Forest Plan had not been in effect. Simulation models for Northern Spotted Owls could be developed to help further explore this system and distinguish between hypotheses [51], perhaps by leveraging existing models that appear to recover empirically-derived demographic patterns [52] or that have been used to address other questions associated with management of Northern Spotted Owls in the Pacific Northwest [53–55]. Likewise, gravity models that incorporate within-site characteristics across the landscape could also potentially help better understand the dynamics of this system and provide novel insights for resource managers [56].

Other factors aside from habitat connectivity and forest condition likely influence genetic structure in Northern Spotted Owls. Recent analyses of long-term dispersal data sets suggest that juvenile spotted owl dispersal distances decreased on the order of ~1 km per year between 1983 and 2012 and that geographic variation in dispersal distances exists, with owls from Oregon demonstrating larger dispersal distances and more long distance dispersal than birds from Washington [49]. The specific mechanisms associated with the net decrease in dispersal distances and geographic variation that have been detected are difficult to identify, however, competition with invasive Barred Owls may be a non-landscape based factor that is contributing to overall patterns [49]. The potential effects of Barred Owls on Northern Spotted Owl dispersal would be consistent with the known negative association between Barred Owls and demographic factors that are also contributing to population declines in the subspecies [7]. Reduced dispersal and declining population sizes can affect genetic structure patterns. Reduced dispersal can lead to a reduction in gene flow, thereby altering genetic connectivity across a landscape. Likewise, reduced population sizes lead to a greater occurrence of inbreeding, which could generate an additional suite of challenges for resource managers to address when implementing conservation measures to mitigate population declines in Northern Spotted Owls.

## Supporting information

S1 Fig Map of dispersal habitat for Northern Spotted Owls from 1986 (see Fig 2) highlighting the approximate locations of the Willamette, Umpqua, and Rogue Valleys. (PDF)
