# Supplementary material for: Isolation by distance versus landscape resistance: Understanding dominant patterns of genetic structure in Northern Spotted Owls (Strix occidentalis caurina)
Source: PLoS One. 2018 Aug 2;13(8):e0201720. doi: 10.1371/journal.pone.0201720 (PMC6072037; doi:10.1371/journal.pone.0201720)
Supplement: S1 Fig — (PDF) [file pone.0201720.s001.pdf]

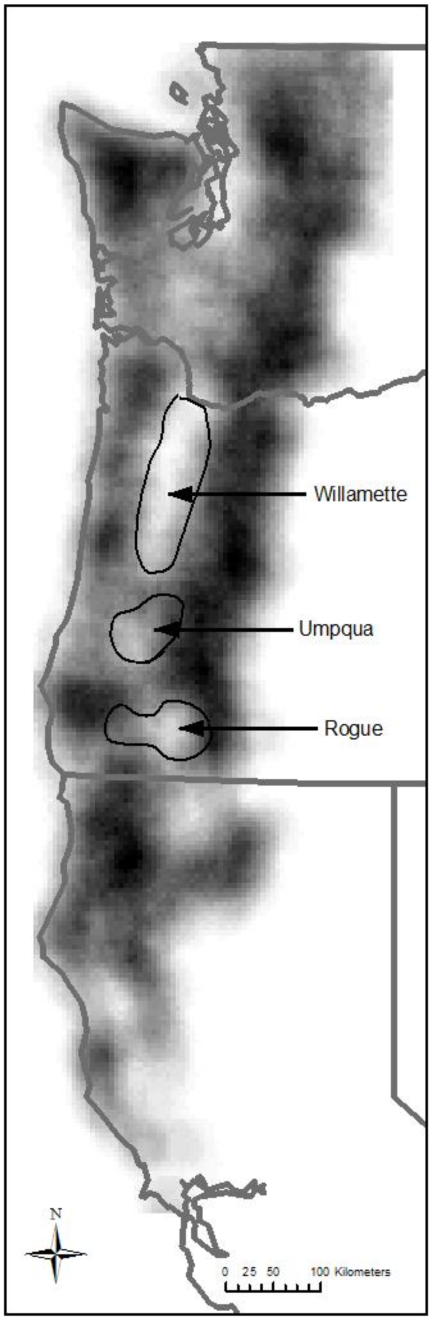

S1 Fig. Map of dispersal habitat for Northern Spotted Owls from 1986 (see Fig 2) highlighting the approximate locations of the Willamette, Umpqua, and Rogue Valleys.
